# Caring for the Critically Ill Adult Congenital Heart Disease Patient

**DOI:** 10.1007/s11886-024-02034-5

**Published:** 2024-04-09

**Authors:** Thomas Das, Penelope Rampersad, Joanna Ghobrial

**Affiliations:** Heart, Vascular and Thoracic Institute, Cleveland Clinic, 9500 Euclid Avenue, Cleveland, OH 44195 USA

**Keywords:** Critical care cardiology, Adult congenital heart disease, Mechanical circulatory support, Intensive care

## Abstract

**Purpose of Review:**

This review aims to discuss the unique challenges that adult congenital heart disease (ACHD) patients present in the intensive care unit.

**Recent Findings:**

Recent studies suggest that ACHD patients make up an increasing number of ICU admissions, and that their care greatly improves in centers with specialized ACHD care. Common reasons for admission include arrhythmia, hemorrhage, heart failure, and pulmonary disease.

**Summary:**

It is critical that the modern intensivist understand not only the congenital anatomy and subsequent repairs an ACHD patient has undergone, but also how that anatomy can predispose the patient to critical illness. Additionally, intensivists should rely on a multidisciplinary team, which includes an ACHD specialist, in the care of these patients.

## Introduction

Due to advances in the recognition and management of congenital heart disease (CHD), the survival of patients in the pediatric population has dramatically increased. Survival into adulthood is now expected for 90% of children diagnosed with CHD, and more adults are estimated to be living with CHD than children [1–4]. As the adult congenital heart disease (ACHD) population continues to grow, the prevalence of hospitalization and intensive care unit admission for these patients has also increased [5]. One Critical Care Cardiology Trials Network study showed that ACHD patients make up approximately 2% of all cardiac intensive care unit (CICU) admissions [6]. Despite their relatively small number, these patients present unique anatomic and physiologic challenges in the adult ICU that the contemporary intensivist should be familiar with. In a single-center study of 138 ACHD ICU admissions, the most common reasons for admission were arrhythmia, hemorrhage, heart failure, and pulmonary disease [7•]. This review aims to discuss practical disease-specific management strategies and general ICU considerations for the ACHD patient.

## General Principles

Successfully caring for the critically ill ACHD patient depends on the practicing intensivist knowing their patient and adjusting their management for personalized care depending on the underlying CHD. Understanding the initial congenital anatomy, as well as the subsequent surgical and transcatheter repairs, is a crucial first step. Reviewing surgical or catheterization reports can lead to key insights on potential triggers for decompensation. If these reports are unavailable, providers must be able to reverse engineer this history from available imaging (whether it be echocardiographic or cross-sectional). In addition to characterizing the cardiac substrate, the baseline end-organ function should be determined; several ACHD patients, particularly those with Fontan circulations, have concomitant pulmonary, renal, hepatologic, and hematologic dysfunction that can further complicate critical illness. Reviewing objective data is necessary, as ACHD patients may not be able to provide the details of their prior history and, at times were misinformed that they were “cured” following childhood surgery [8].

Given the highly multifaceted nature of managing the critically ill ACHD patient, it is imperative that intensivists seek the insight and guidance of a multidisciplinary team, especially the ACHD specialist. Depending on the underlying anatomy and illness, this can include specialists in cardiac surgery, interventional cardiology, electrophysiology, imaging, advanced heart failure, and non-cardiac specialists such as hepatology, nephrology, and pulmonology. In all cases, intensivists should seek the input and collaboration of a provider with specific expertise in adult congenital heart disease [9, 10]. ACHD patients cared for in centers with ACHD expertise have better outcomes than those patients in centers without this expertise, including improved mortality [11]. If a given center is unable to provide this level of expertise, patient transfer to a more appropriately equipped center should be explored if the patient can be safely transported [12].

Whether ACHD patients are best cared for in a pediatric or adult intensive care unit is controversial. While providers in a pediatric setting are likely more familiar with the underlying anatomic underpinnings of CHD, those in an adult setting may have more experience in managing the multiple comorbidities often associated with adulthood. This decision should be made on a case-by-case basis, as whichever setting has the resources that best match the patient’s needs is likely most appropriate.

## Hemodynamic Assessment

An accurate and thorough hemodynamic assessment can be vital in identifying etiologies of critical illness and monitoring response to therapy in ACHD patients. An assessment of blood pressure, peripheral oxygenation, cardiac output, systemic resistance, and pulmonary resistance should be obtained, and compared to baseline “well” evaluations; while a systolic blood pressure less than 90 mmHg or oxygenation saturation of less than 88% may identify critical illness in some patients, these values could represent baseline conditions in some ACHD anatomies [13]. Furthermore, complex congenital anatomy can present several challenges in otherwise routine hemodynamic monitoring. This is particularly true in measuring the blood pressure, as both non-invasive and invasive blood pressure readings can be falsely low in the setting of a prior classic Blalock-Thomas-Taussig (BTT) shunt due to anastomosis of the subclavian and pulmonary arteries [14]. If an invasive arterial line is needed, the radial artery of the arm not affected by the shunt or a femoral artery should be used. A similar situation may be encountered in patients with severe coarctation, where the initial coarctation site and the subsequent repairs can affect accurate blood pressure assessment. In patients with a shunt, such as a patent ductus arteriosus (PDA) and Eisenmenger syndrome, care must be given to obtaining saturations pre- and post-shunt, in both upper and lower extremities to assess pre- and post-ductal saturations and estimate shunting.

While invasive real-time hemodynamic monitoring using a pulmonary artery catheter is returning to prominence in the CICU, this can be challenging and potentially contra-indicated in certain congenital anatomies [15]. In some scenarios, it can be misleading; in patients with pulmonary atresia with multi-level branch pulmonary stenosis (PS), a wedge pressure may be inaccurately elevated at the site of PS, and a direct left ventricular (LV) end-diastolic pressure (EDP) is the more accurate assessment of LV preload. Furthermore, in severe right ventricular (RV) to pulmonary artery (PA) conduit stenosis, an elevated RV systolic pressure is not indicative of pulmonary hypertension, and crossing a critically stenotic conduit may be both difficult and lead to further reduction in pulmonary blood flow or hemodynamic compromise. Given the higher risk of thrombosis, patients with Fontan conduits should not have an indwelling central venous or pulmonary artery catheter placed that remains in the Fontan conduit; if one is left in situ, one should strongly consider systemic anticoagulation [16]. Given the importance of maintaining adequate preload in the Fontan circulation, a simple central venous catheter that terminates in the SVC may be considered for a short period of time when large fluid shifts are expected, such as during a large-volume paracentesis or aggressive diuresis [17]. If needed, right heart catheterization should be fluoroscopically guided with removal of the catheter at the conclusion of the procedure.

## Cardiopulmonary Resuscitation

Special note should be paid to the challenges in performing CPR in patients with Fontan circulations. In these patients, due to the lack of a sub-pulmonic ventricle, during chest compression, blood can flow backward in the Fontan conduit, limiting the effectiveness of CPR. This often leads to inadequate pulmonary blood flow during chest compressions, leading to decreased oxygenation and preload to the systemic ventricle, and subsequently decreased cardiac output and end-organ perfusion. Central venous pressure rises significantly, worsening cerebrovascular congestion and increasing the risk of neurological injury. These considerations do not mean that CPR should not be performed or shortened in these patients, but rather that  the transition from CPR to emergent mechanical support may need to be expedited. Patients with dextrocardia may also have a more rightward point of optimal chest compressions and defibrillation than those with levocardia [18].

## Pulmonary Disease and Mechanical Ventilatory Support

ACHD patients often have underlying pulmonary disease, such as restrictive lung disease from multiple prior cardiac surgeries or cardiomegaly, diaphragmatic paralysis due to phrenic nerve injury, and airway disease from multiple intubations [19]. Additionally, pulmonary hypertension can be present in up to one-third of patients with ACHD. Due to low pulmonary reserve, concomitant pulmonary hypertension, and alterations in pulmonary vascular blood flow, ACHD patients are often uniquely susceptible to pulmonary insults [20]. In patients with Fontan conduits dependent on passive blood flow through the pulmonary vascular bed, conditions in which pulmonary vascular resistance acutely rises can lead to a dramatic drop in preload of the single ventricle and subsequent hemodynamic collapse [21, 22]. This includes pneumonia, pulmonary embolism, pneumothorax, and excessive positive end-expiratory pressure (PEEP) in the setting of mechanical ventilation. Diagnosis of pulmonary embolism can be particularly nuanced in Fontan patients, as differential distribution of contrast through the Fontan and Glenn conduits can lead to false positive diagnosis; this can be ameliorated by dual contrast injections via the upper and lower limbs or by protocoling the imaging to allow adequate contrast circulation for complete opacification through the pulmonary vasculature. At times invasive pulmonary angiography is indicated to definitively diagnose and potentially intervene in pulmonary emboli [23].

Tailoring oxygenation support can be challenging in ACHD patients with cyanosis or residual shunts, as a peripheral oxygenation saturation of less than 90% may represent their baseline. Worsening oxygen saturation below baseline, worsening respiratory rate, or lactic acidosis can all signal relative hypoxemia necessitating supplemental oxygenation. Conversely, intensivists should recognize that targeting a “normal” oxygenation in these patients before de-escalating support is detrimental and should instead individualize O2 goals to the individual patient.

Just as the number of ACHD patients admitted to the ICU has increased over recent years, so, too, has the number of ACHD patients requiring mechanical ventilation [24]. Intubation can have severe detrimental hemodynamic effects on congenital anatomies; an acute drop in preload associated with induction can cause hemodynamic collapse in patients with Fontan circulation.  Excessive PEEP, which reduces preload and increases afterload, can worsen failing sub-pulmonic ventricles in the setting of pulmonary hypertension or Tetralogy of Fallot (ToF). The increase in pulmonary afterload from mechanical ventilation could acutely worsen an intra-atrial right to left shunt, causing profound hypoxia, which in turn can further exacerbate pulmonary vascular constriction [25]. Conversely, the decrease in LV afterload and preload associated with PEEP can be beneficial in patients with predominantly systemic ventricular failure. Consequently, intubation of ACHD patients should be performed by providers familiar with congenital anatomy and cardiac anesthesia. Interestingly, one single-center study showed that ACHD patients have a relatively low incidence of difficult endotracheal intubation when compared to non-ACHD patients, possibly due to a different age and weight profile compared to the general population [26] .

## Hepatic Disease

Patients with congenital heart disease often have comorbid liver dysfunction due to venous congestion, decreased cardiac output, hypoxemia, or ischemic injury to the hepatic parenchyma. This is especially common in patients with right-sided lesions (such as ToF or Ebstein anomaly), or in single ventricle physiology palliated with Fontan circulation [27]. For patients who underwent surgery before 1992, viral hepatitis infection from a blood transfusion should also be considered an etiology of liver disease [28]. As ACHD patients age and develop, the comorbidities of adulthood, cirrhosis due to alcohol use, or non-alcoholic fatty liver disease has also been observed in this population. Regardless of the etiology, patients with ACHD and liver disease suffer from coagulopathies and aberrant hemodynamics that can further complicate their care in the ICU. Fontan-associated liver disease can be particularly challenging to recognize, as these patients do not exhibit the elevation in transaminases or transhepatic gradient seen in other forms of liver disease [29]. However, the presence of FALD can often complicate an advanced therapy evaluation for a critically ill Fontan patient, and candidacy for both liver and heart transplantation would need to be evaluated [30]. Another consideration in critically ill patients with failing Fontan physiology is severe protein-calorie malnutrition secondary to protein losing enteropathy (PLE), characterized by hypoalbuminemia, enteric protein loss, diarrhea, and edema. Several pathophysiological mechanisms have been proposed to explain this phenomenon, including chronic inflammation and chronic central venous congestion. It is associated with significant morbidity and mortality in this population, and is an indication for transplant evaluation.

## Renal Disease

Chronic kidney disease is common in the ACHD population and is associated with an increased risk of adverse outcomes [31]. This is especially true in ACHD patients with cyanosis, as more than 65% of these patients have some degree or renal dysfunction [32]. Quantification of glomerular filtration rate should ideally be done using a cystatin C–based method, as this has been shown to more accurately predict clinical events than creatinine-based methods in ACHD patients [33]. Management of renal dysfunction and acute kidney injury is largely similar in ACHD patients to that in non-ACHD cardiac patients, on optimizing pre-load and perfusion, decreasing renovascular congestion, and if needed, initiation of continuous renal replacement therapy. Notably, patients with a single ventricle physiology often do not tolerate the large volume shifts associated with hemodialysis, complicating the transition from continuous to intermittent dialysis.

## Hematological Disease

ACHD patients are uniquely prone to both clotting and hemorrhagic complications, depending on their underlying anatomy. Patients with cyanotic heart disease and Eisenmenger syndrome exhibit secondary erythrocytosis, platelet dysfunction, decreased fibrinogen, hemostatic abnormalities, and endothelial dysfunction. This constellation of risk factors uniquely predispose Eisenmenger patients to both spontaneous bleeding events (the most dire being hemoptysis and intrapulmonary hemorrhage) and thromboembolic events, which can lead to cerebrovascular injury due to right to left shunting [34]. Therapeutic phlebotomy should only be pursued in the presence of severe hyper-viscosity symptoms (such as acute thrombosis and neurological injury) despite adequate hydration. Fontan patients are also at increased risk of thrombus formation due to sluggish circulation throughout the passive sub-pulmonic conduit; this can lead not only to Fontan failure but also paradoxical emboli and stroke with residual fenestrations or venovenous collaterals.

Acute blood loss anemia in patients with baseline erythrocytosis can also be difficult to recognize, as a significant drop in hemoglobin or hematocrit could still fall in the “normal” lab range. Screening for iron deficiency and repleting iron stores improves oxygen-carrying capacity. Assessing for changes in the hemodynamics or end-organ perfusion can help guide resuscitation with blood products in these patients.

## Primary Cardiac Disease

### Arrhythmia

Both tachyarrhythmia and bradyarrhythmia are commonly encountered in the ACHD population and can often lead to ICU admission. Intra-atrial reentry tachycardia (IART), in which the scarred atria promote the development of macro re-entry pathways independent of the cavo-tricuspid isthmus, is the most commonly encountered atrial arrhythmia in ACHD patients (though atrial fibrillation and typical flutter are also common) [35]. Atrioventricular reentrant tachycardia and Wolff-Parkinson-White syndrome should also be considered in patients with Ebstein anomaly, Ebstein-like tricuspid valves, or congenitally corrected transposition of the great arteries (ccTGA) ebstinoid valves [36]. As the loss of AV synchrony and the atrial kick can cause hemodynamic collapse in an already tenuous congenital anatomy (such as cyanotic disease or single ventricle physiology), atrial arrhythmias should be treated expeditiously with a rhythm control strategy (either medically or through electrical cardioversion). Notably, CHA2DS2-VASc scores do not accurately predict the risk of thromboembolism in ACHD patients, and ACHD patients with atrial arrhythmia are typically anticoagulated [37]. Ventricular tachycardias are commonly seen in patients with ToF, either due to underlying myopathic processes or isthmus defined by surgical scar and patch material. While ToF patients may have implanted cardiac defibrillators (ICDs), they also have a high burden of inappropriate shocks due to atrial arrhythmias [38]. A careful device interrogation should be performed before incorrectly diagnosing such a patient with electrical storm. In the setting of incessant tachyarrhythmia refractory to medical therapy, catheter-based ablation procedures can be considered; these should be performed by electrophysiologists with expertise in congenital anatomy.

Bradyarrhythmias in congenital heart disease can be categorized into congenital abnormalities of the conduction system, acquired bradyarrhythmias following surgical or percutaneous intervention, or mixed causes [39]. Patients with ccTGA are at high risk of progressive heart block due to anatomic distortion of the conduction system, with mean age of heart block occurrence of 18 years old. Additionally, many surgical repairs of CHD can lead to sinus node dysfunction, including atrial switch procedures in D-transposition of the great arteries (i.e., Mustard and Senning surgeries) and repair of sinus venosus ASDs. Infrequently, transcatheter membranous VSD device closure can lead to heart block. Depending on the presence of a sub-pulmonic ventricle and adequate venous access, placement of an emergent temporary transvenous pacemaker may not be possible in the ICU setting (though emergent placement of a transvenous pacemaker in a Fontan baffle has been reported) [40]. In these cases, pharmacologic methods to increase chronotropy and even transcutaneous pacing may need to be pursued to bridge the patient to definitive surgical epicardial pacemaker placement.

### Heart Failure

Acute decompensated heart failure (ADHF) is a leading cause of death in ACHD patients and represents a significant burden of ACHD ICU admissions [7•, 41]. Early stages of decompensation can sometimes be difficult to identify in ACHD patients who have lived with a limited functional capacity since childhood; consequently some patients may not present to care until they are in extremis. Medical heart failure therapy in a biventricular circulation with a morphological systemic left ventricle does not differ much from that in the acquired heart failure population. Guideline-directed medical therapy (including beta blockers, ACEi/ARB/ARNI, SGLT2 inhibitors, and mineralocorticoid antagonists) should be pursued [42•]. However, patients with single ventricle physiology and systemic right ventricles may not respond the same to heart failure medications and there has been inconclusive evidence of benefit.

When assessing the ACHD patient in ADHF, identifying potentially correctible lesions can be crucial in guiding care and delaying the need for advanced therapies; these include pulmonary valve regurgitation in tetralogy of fallot, baffle obstruction or leak in d-TGA with atrial switch, systemic tricuspid valve regurgitation in ccTGA and patients with Norwood corrections, and Fontan conduit obstructions [13, 43]. Transcatheter interventions can sometimes correct these lesions, and reverse the underlying shock state [44, 45]. As ACHD patients age and accrue risk factors for atherosclerosis, acute coronary syndromes should also be considered correctable triggers for ADHF in the ACHD population [46, 47]. Anomalous coronary arteries, myocardial bridges, and coronary compression from an aneurysmal pulmonary artery or oversized prosthetic pulmonary valve can also lead to myocardial ischemia and, ultimately, shock in certain ACHD patients [48, 49].

Advanced heart failure is a common end point in patients with an underlying myopathic process, such as those with systemic right ventricles, severe pulmonary hypertension with failure of the sub-pulmonic ventricle, tetralogy of fallot, and single-ventricle physiologies with Fontan palliation. As with acute decompensated heart failure-cardiogenic shock (ADHF-CS) in the non-ACHD population, initial medical therapy focuses on optimizing volume status and supporting blood pressure. In right heart failure lesions, attention should be made to maintain adequate preload without over-distending a failing RV, as well as reducing the RV afterload with PVR reduction and maintaining adequate SVR for renal perfusion. In Fontan circulations, a higher central venous pressure should be targeted as filling of the single ventricle is particularly preload sensitive [50]. Vasoactive medications should be selected based on the underlying hemodynamic profile and insult; norepinephrine or epinephrine is generally a reasonable starting choice; however, both can increase PVR and promote arrhythmias. Vasopressin could be considered in patients with pulmonary hypertension given selective effects on peripheral vasoconstriction without significantly affecting PVR [51]. In select patients with single-ventricle physiology, a combination of an inotropic agent (such as milrinone or dobutamine) to improve single-ventricular function plus a pulmonary vasodilator (such as inhaled nitric oxide, iloprost, or epoprostenol) to improve flow through the “bottle neck” of the pulmonary vascular bed can improve cardiac output. While potentially effective, consideration should be made to hypotension with this combination.

In patients with worsening ADHF-CS refractory to medical therapy, temporary mechanical circulatory support (tMCS) should be considered. From 2004–2014, the use of tMCS in ACHD patients has increased by over 250%; however, this has not been accompanied by improved in-hospital mortality, which can be as high as 58–60% [52]. When deploying tMCS in ACHD patients, determining which ventricles need to be supported and the vascular access for delivering the chosen devices are both key considerations. As these patients have often undergone several vascular access procedures throughout their lifetimes, scarring around access sites, or chronic occlusions of vessels, can complicate deployment of tMCS. Univentricular systemic ventricle support devices (such as the intra-aortic balloon pump or impella percutaneous ventricular assist device) have both been deployed in patients with congenital heart disease [53–55]. The use of right (or sub-pulmonic) ventricular support devices is still growing in the general ICU population, though it may have particular utility in the ACHD population that often suffers from sub-pulmonic ventricular failure [56–58]. In situations where biventricular support and oxygenation support is needed, veno-arterial extracorporeal membrane oxygenation (VA-ECMO) should be considered. One retrospective study showed that patients with ACHD who required ECMO had longer lengths of stay and suffered more complications than those without ACHD [59]. When deploying VA-ECMO, reviewing the congenital anatomy to understand aortic and cavo-atrial connections is key to ensuring that arterial and venous cannula are well positioned. Patients with a Fontan undergoing peripheral VA-ECMO cannulation require drainage cannulae in both the SVC and IVC (VVA-ECMO) [60]. However, this approach may lead to stasis in the Fontan conduit. Another potential strategy is a VAV configuration with oxygenated blood return to the upper part of the body, ensuring adequate oxygenation and promoting more flow in the Fontan conduit.

Prior to deploying tMCS in all critically ill ACHD patients, a potential exit strategy should be identified. This can include bridge to intervention (in the setting of a potentially reversible valvular or coronary insults), bridge to recovery (in the setting of decompensated heart failure expected to improve with medical therapies), or bridge to durable mechanical support or transplant.

### Advanced Heart Failure Therapies

The decision to pursue advanced heart failure therapies (such as durable mechanical circulatory support or organ transplant) in the critically ill ACHD patient should be made in with the multidisciplinary input of specialists in congenital heart disease, critical care, advanced heart failure, and congenital cardiac surgery. Patients with CHD can be disadvantaged by the United Network for Organ Sharing (UNOS) heart transplant allocation criteria, and on average stay longer on the transplant waitlist and experience higher waitlist mortality [61–63]. Notably since the 2018 UNOS allocation revision for transplantation, there has been an increase in the use of tMCS to shorten the wait time for organ transplant. Durable mechanical support, in the form of a durable ventricular assist device (VAD) or total artificial heart (TAH), can be considered an exit strategy from temporary mechanical support [64]. Again, device choice is determined by the underlying anatomy and physiology. VADs have been implanted to support systemic right ventricles, though this often requires an adequately RV volume, the resection of trabeculae, and alternative positioning of the inflow cannula [65]. VADs have also been implanted to support those with Fontan palliations, both as a bridge to transplant and as a destination therapy [66]. A study of the Interagency Registry for Mechanically Assisted Circulatory Support (INTERMACS) showed that ACHD patients with LVADs have similar survival to non-ACHD patients. In contrast, those with TAH or biventricular assist devices had worse outcomes [67]. Cavo-pulmonary assist devices specifically designed for Fontan circulations are currently under development, though they are currently in the in-vitro and in-vivo model phase [68].

The long heart transplant waitlist times for ACHD patients are multifactorial, including high rates of sensitizations from prior surgeries and blood transfusions and lower urgency listings. [69] In order to prioritize ACHD patients, UNOS has published guidance on exception requests for ACHD patients [70]. While ACHD patients who undergo heart transplant have twice the 30-day mortality of non-ACHD patients, the patients who survive past one year have superior survival to non-ACHD patients [71, 72•].

### Neuropsychiatric Considerations

ACHD patients are at high risk of neuropsychiatric pathology that is often exacerbated in the critical care setting [73]. Both structural neurologic disease (such as prior stroke and frontal lobe injury) and psychiatric disease (such as depression and anxiety) have been observed at high rates in this population [74]. This can complicate sedation in the context of mechanical ventilation as well as complex goals of care decision-making. Involving a patient’s family and support system can be beneficial in these settings. Importantly, ACHD patients are also at risk of acute neurological insult due to increased risk of stroke and systemic thromboembolism [75]. This is particularly true in patients with right to left shunts and in cyanotics; in these patients, the use of bubble filters on peripheral IVs can mitigate the risk of paradoxical embolism.

## Conclusions

The care of the critically ill adult congenital heart disease patient is complex and requires the input of a multidisciplinary team. As this population continues to grow and age, it is incumbent on intensivists to recognize the unique challenges in caring for this population, and work with congenital heart disease specialists to support these patients through critical illness.
